# Editorial: Pathogenic and symbiotic bacteria in ruminants: antimicrobial resistance and microbial homeostasis

**DOI:** 10.3389/fvets.2023.1355704

**Published:** 2024-01-08

**Authors:** Yi Yang, Jilei Zhang, Heba Sayed El-Mahallawy

**Affiliations:** ^1^Jiangsu Co-innovation Center for Prevention and Control of Important Animal Infectious Diseases and Zoonoses, College of Veterinary Medicine, Yangzhou University, Yangzhou, China; ^2^International Corporation Laboratory of Agriculture and Agricultural Products Safety, Yangzhou University, Yangzhou, China; ^3^Division of Gastroenterology and Hepatology, Department of Medicine, University of Illinois at Chicago, Chicago, IL, United States; ^4^Department of Zoonoses, Suez Canal University, Ismailia, Egypt

**Keywords:** ruminant, pathogenic bacteria, symbiotic bacteria, antimicrobial resistance, microbial homeostasis, microbiome, dysbiosis

Ruminants share a profound connection with human beings. They have the remarkable ability to harness rumen microorganisms, facilitating the fermentation process and converting plant proteins into high-quality animal proteins. This process yields a significant supply of meat and dairy products indispensable to our lives. The diversity and abundance of rumen microorganisms indicate not only the host's digestive and metabolic capacity but also its health status. During the long process of evolution, ruminants have adapted to environmental changes by modifying the types and abundance of microorganisms in their rumen, thereby optimizing energy utilization (Yang et al.).

Nonetheless, alongside the beneficial symbiotic bacteria in the rumen, numerous pathogenic bacteria pose a grave threat to the wellbeing of ruminant animals. Brucellosis is a zoonosis of significant public health and economic importance that is endemic in ruminants worldwide. Compared with large farmed ruminants (cattle, zebu, and buffalo), small farmed ruminants (goats and sheep) infected with *Brucella melitensis* or *B. ovis* pose a greater threat to humans, especially in African countries (Hussen et al.).

The dairy market primarily comprises milk from cows, goats, water buffaloes, and camels, with cow's milk taking the lead in consumption. However, goat, water buffalo, and camel milk stand out due to their distinctive nutritional components, making them particularly suitable for specific demographic groups. Mastitis is the most common, costly, and important disease in the dairy industry. Up to now, more than 150 species of pathogenic bacteria have been identified in the raw milk of animals with mastitis. *Staphylococcus aureus* is frequently isolated in many countries (1). For example, Wang K. et al. have identified a high pooled prevalence of *S. aureus* (36.23%) in China from 2000 to 2020. Although *S. aureus* has been well documented and recognized as a significant mastitis-causing organism in cows, its molecular characteristics and pathogenicity in water buffaloes are largely unknown. A recent epidemiological study conducted in Guangdong province, China, revealed that *S. aureus* is the third most prevalent pathogenic bacteria in water buffaloes with subclinical mastitis, and its isolation frequency is lower than *Escherichia coli* and coagulase-negative staphylococci (Zhang et al.).

Due to the frequent occurrence of mastitis and repeated use of antibiotics (administered during the lactation, or at dry-off) (Okello et al.), bacteria isolated from raw milk are gradually developing antimicrobial resistance. In recent years, the antibiotic resistance phenotypes and genotypes of mastitis pathogens, as well as the development of new antibiotic replacement therapies, have become prominent areas of study. For example, Toquet et al. has reported the *in vivo* antimicrobial potential of lactic acid bacteria (a kind of probiotics) in the treatment of contagious agalactia caused by *Mycoplasma agalactiae*. Although hundreds of pathogenic bacteria are associated with mastitis, studies on antibiotic resistance are mainly focused on *S. aureus, Escherichia coli* and *Streptococcus*. A high prevalence of *S. aureus* has been reported to be resistant to penicillin G, ampicillin, or amoxicillin. In contrast, a low prevalence of isolates was resistant to trimethoprim-sulfamethoxazole or gentamycin (2). *E. coli* and *Streptococcus* are also major mastitis-causing pathogens in dairy cows. Multidrug-resistant (acquired resistance to ≥ three classes of antimicrobials) *Streptococcus* can be frequently isolated from raw milk of cows with mastitis, with the presence of antibiotic resistance genes and virulence genes (3, 4).

In addition to mastitis, veterinarians should pay much attention to respiratory and digestive diseases caused by pathogenic bacteria infection. Diarrhea can be caused by different kinds of pathogens, including bacteria (*E. coli* K99/ O157 and *Salmonella enteritidis*), viruses (bovine viral diarrhea virus, bovine and ovine rotavirus), and parasites (*Cryptosporidium* sp. and *Coccidium* sp.) (Wang D. et al.). Respiratory disease can result in slow weight gain in beef cattle and sheep, causing considerable financial losses for beef and lamb producers. Airway microbiotas enriched with probiotics (such as *Lactobacillus*) are associated with good respiratory health. On the contrary, microbiotas enriched with recognized pathogenic bacteria (*Klebsiella pneumoniae* and *Pasteurella multocida*) are related to respiratory diseases (5). The threat of respiratory and digestive disease for cattle, sheep and goat operations is exacerbated by increasing prevalence of antimicrobial resistance in pathogenic bacteria (Carter et al.).

*Anaplasma*, a kind of tickborne pathogen, can cause anaplasmosis in ruminants. Previous studies have demonstrated the presence of *A. marginale, A. ovis, A. platys*, and *A. phagocytophilum* in ruminants, and *A. marginale* (Mahmoud et al.) and *A. phagocytophilum* can be frequently detected (6, 7). It is worth noting that *A. phagocytophilum* is a zoonotic pathogen that can cause human granulocytic anaplasmosis (HGA). HGA is characterized by sustained high fever and a decrease in white blood cells and platelets. Its clinical manifestations primarily include overall discomfort, fatigue, headache, muscle soreness, and symptoms like nausea, vomiting, loss of appetite, and diarrhea. Misdiagnosis is common due to the similarity of its symptoms to certain viral infectious diseases. In severe cases, it can lead to multiple organ dysfunction, including the heart, liver, and kidneys, potentially resulting in fatal outcomes.

In conclusion, the contributions to this Research Topic expand our understanding of the distributions and characteristics of pathogenic and symbiotic bacteria in ruminants, providing valuable insights to improve our ability to safeguard the health of these animals.

## Author contributions

YY: Writing – original draft, Writing – review & editing. JZ: Writing – review & editing. HE-M: Writing – review & editing.
